# Comparing the Selection and Placement of Best Management Practices in Improving Water Quality Using a Multiobjective Optimization and Targeting Method

**DOI:** 10.3390/ijerph110302992

**Published:** 2014-03-11

**Authors:** Li-Chi Chiang, Indrajeet Chaubey, Chetan Maringanti, Tao Huang

**Affiliations:** 1 Department of Civil and Disaster Prevention Engineering, National United University, Miaoli 36003, Taiwan; E-Mails: lchiang@nuu.edu.tw (L.-C.C.); r99622021@ntu.edu.tw (T.H.); 2 Department of Earth, Atmospheric, and Planetary Sciences; Department of Agricultural and Biological Engineering, Purdue University, 550 Stadium Mall Drive, West Lafayette, IN 47907, USA; E-Mail: ichaubey@purdue.edu; 3 Risk Modeling Unit, Zurich Financial Services Ltd., Mythenquai 2, Zurich 8002, Switzerland; E-Mail: chetan.maringanti@zurich.com

**Keywords:** best management practice, nonpoint source pollution, multiobjective optimization, genetic algorithm, Soil and Water Assessment Tool

## Abstract

Suites of Best Management Practices (BMPs) are usually selected to be economically and environmentally efficient in reducing nonpoint source (NPS) pollutants from agricultural areas in a watershed. The objective of this research was to compare the selection and placement of BMPs in a pasture-dominated watershed using multiobjective optimization and targeting methods. Two objective functions were used in the optimization process, which minimize pollutant losses and the BMP placement areas. The optimization tool was an integration of a multi-objective genetic algorithm (GA) and a watershed model (Soil and Water Assessment Tool—SWAT). For the targeting method, an optimum BMP option was implemented in critical areas in the watershed that contribute the greatest pollutant losses. A total of 171 BMP combinations, which consist of grazing management, vegetated filter strips (VFS), and poultry litter applications were considered. The results showed that the optimization is less effective when vegetated filter strips (VFS) are not considered, and it requires much longer computation times than the targeting method to search for optimum BMPs. Although the targeting method is effective in selecting and placing an optimum BMP, larger areas are needed for BMP implementation to achieve the same pollutant reductions as the optimization method.

## 1. Introduction

Nonpoint source (NPS) pollution from agricultural watersheds has become one of the major water quality concerns [1,2]. For example, more than 70% of the delivered nitrogen (N) and phosphorus (P) in the Mississippi River Basin are contributed from the adjacent agricultural lands and these increased nutrient fluxes are linked to seasonal hypoxia in the northern Gulf of Mexico [3]. Excessive fertilizer usage on tea fields in Taiwan was identified as the major source of ammonia, which can lead to eutrophication [4]. Agricultural practices not only determine the level of food production, but also the state of the global environment, including water quality, soil quality, and species composition [5]. Intensive agricultural practices are considered sources of significant amounts of nutrients, especially nitrogen (N) and phosphorus (P), pesticides, fecal bacteria and sediment to receiving water bodies [6,7], reducing the ability of ecosystems to provide goods and services [5]. Usually in an agricultural watershed with concentrated animal production operations, improper usage of manure with commercial fertilizers could result in excessive nutrient losses from the fields to the receiving water bodies [8,9]. Sediment losses from top soil containing relatively large amounts of nutrients can threaten water quality and decrease the productive capacity of the land [10]. 

The adverse impacts from agricultural areas can be controlled by implementing best management practices (BMPs) to reduce source or retard pollutant transports in a watershed. Many studies have used simulation models to evaluate BMP effectiveness and determine the optimum BMPs to improve water quality at the farm level [11] and at a watershed level [12,13,14,15,16]. However, considering resource constraints, it is not possible to implement BMPs in every candidate location in a watershed. Besides, certain critical areas in a watershed may contribute disproportionally large amounts of pollutants in a watershed. Pionke *et al.* [17] concluded that up to 90% of the annual phosphorus loads were contributed by approximately 10% of the watershed in the Brown catchment (Pennsylvania, USA) Therefore, several methods have been developed to select and place cost-effective BMPs in a watershed. Those methods can be categorized into plan- or performance-based methods [18]. Plan-based methods are mainly used to assign BMPs based on the identification of critical areas in a watershed. However, interactions among BMPs on pollutant reduction are typically not considered in plan-based methods. The performance-based method incorporates simulation models to evaluate the cost-effectiveness of selected BMP combinations based on their individual performance and cost. 

Targeting is a plan-based method to place BMPs in critical source areas which contribute a disproportionate amount of NPS pollutants. Many studies have been conducted to identify critical source areas and to estimate the improvement of water quality due to implementation of selected BMPs in those critical regions [18,19,20,21]. Because spatial interactions among BMPs are not considered in establishing a targeting strategy, a BMP that is selected based on certain targeting strategy may or may not be the most cost-effective BMP for the watershed. In contrast, optimization is a performance-based method that considers the effectiveness and cost of various BMPs, evaluates numerous BMP scenarios and incorporates the impacts of BMP interactions in assessing the cost-effectiveness of BMP scenarios. Many studies have combined the genetic algorithm (GA) and NPS prediction models to optimize the BMP selection and placement in a watershed [22,23,24,25,26,27]. Most of the previous work has focused on using a single objective function which combines both BMP effectiveness and cost [23], sequentially optimizing two objective functions separately [24,25] or optimizing two objective functions of BMP effectiveness and cost simultaneously [22]. Zare *et al.* [28] applied the Non-dominated Sorting Genetic Algorithm II (NSGA-II) optimization technique to derive the optimal tradeoff curve simultaneously between three objectives: reducing cost of BMP implementation, maintaining runoff quality, and minimizing runoff volume. Cost of BMPs was estimated based on the volume (Rain barrel and Bio-retention) or the areas (Porous pavement).

Optimization studies related to selection of BMPs have traditionally used cost minimization as an objective function. Cost as an optimization function does not ensure that the watershed areas under BMPs are also minimized. Many researchers have indicated that a relatively small portion of a watershed contributes a larger amount of pollutants. BMPs are generally targeted in those high-risk watershed areas. To enhance the effectiveness of BMPs, achieving the same pollutant reductions with less areas should be considered. In order to compare the watershed areas that needs to have BMPs under targeting and optimization options to produce similar water quality benefits, we used the watershed area as one of the objective functions in this study. Therefore, the overall goal of this study was to compare the selection and placement of optimal BMPs using an optimization model with various BMP options and a targeting method for achieving a high level pollutant reduction with BMP implementation in a small portion of the pasture lands. The hypotheses we tested were as follows (1) selection and placement of BMPs from different sets of BMP options using a genetic algorithm (GA) optimization tool can result in different water quality improvements; (2) Limiting the BMP options to the BMPs which have a greater pollutant reduction rate can assist the optimization tool to allocate BMPs more effectively. We used the multiobjective optimization model developed by Maringanti *et al.* [22]. This optimization model incorporates a BMP tool which replaces the requirement of dynamic linkage with a hydrologic model (Soil and Water Assessment Tool, SWAT) in the BMP optimization architecture. The BMP tool is a database that contains quantitative information of BMP effectiveness in reducing pollutant losses for given land use. The two objective functions optimized in this study were minimizing the pollutant loads and minimizing the areas for BMP implementation in the watershed.

## 2. Materials and Methodology

### 2.1. Study Site

**Figure 1:**
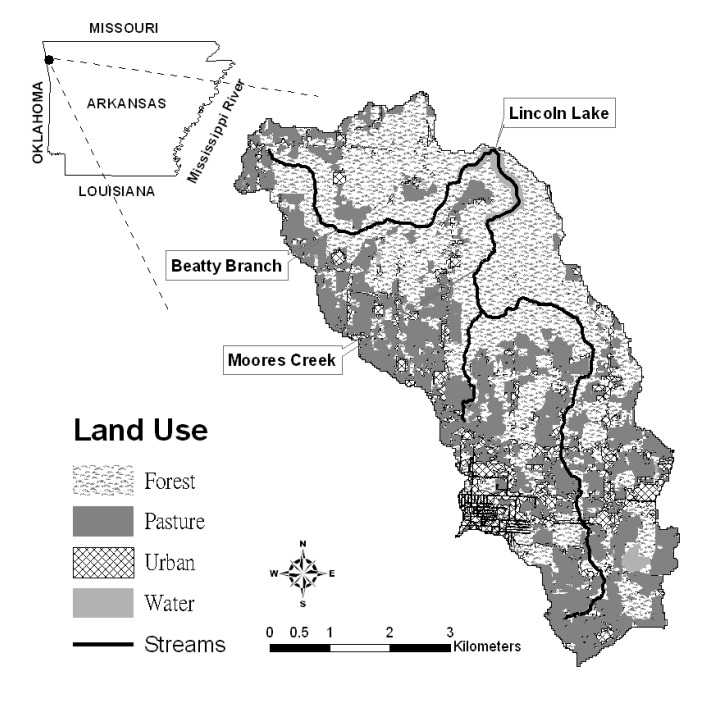
Location of Beatty Branch, Moores Creek, land-use distribution and the gauging stations in the Lincoln Lake watershed.

This study was conducted in the Lincoln Lake CEAP watershed, a 32 km^2^ agricultural watershed within the Illinois River basin located in Northwest Arkansas and Eastern Oklahoma (Figure 1). The average slope in the watershed is 6%. The elevation ranges from 365 to 487 m with a mean elevation of 429 m. The major soil series in the watershed are Enders gravelly loam, Hector-Mountainburg gravelly fine sandy loam, Captina silt loam and Linker loam, which account for 23%, 21%, 13% and 12% of the entire area, respectively. An average annual precipitation of 1,231 mm was observed during 1990–2002 with the highest average monthly precipitation (158.3 mm) in April and the lowest average monthly precipitation (74 mm) in January. The average maximum and minimum temperature during 1990–2002 were 20.1 °C and 8.7 °C, respectively. Excessive nutrient losses due to improper litter application and grazing activity on pasture lands has been one of the main environmental issues in the watershed. The measured total phosphorus (TP) concentration at the Illinois River near the Arkansas-Oklahoma border was about 0.4 mg/L [29].

Moores Creek and Beatty Branch are the two major tributaries in the Lincoln Lake watershed representing 21 and 11 km^2^ of the watershed area, respectively. The watershed has a mixed land use with pasture, forest, urban residential, urban commercial and water representing 35.8%, 48.6%, 11.9%, 1.5% and 2.2% of the watershed area, respectively (Figure 1). The pasture land use area has decreased from 43% to 36% primarily due to increasing urbanization in the watershed since 1994 [30]. Pasture fields in the watershed have numerous poultry, beef, and dairy cattle production facilities. Excessive litter and manure application for perennial forage grass production in the watershed have been shown to increase surface and ground water pollution due to increasing losses of sediment, nutrients and pathogens [31]. Since 1994, BMPs implemented in the watershed have increased from 1% to 34% of the watershed area, representing 53% of total pasture areas in the watershed in 2004. 

### 2.2. SWAT Model Development

The Soil and Water Assessment Tool (SWAT, version SWAT 2009), was used to estimate the effectiveness of various BMP combinations in reducing pollutant losses in a previous study [32]. The SWAT 2009 has the abilities to simulate dynamic land use changes and has improved routines for simulating vegetated filter strips. The model can predict long-term impacts of land use and management on water, sediment and agricultural chemical yields at different scales in a mixed land use watershed [33,34]. More than 250 peer-reviewed journal articles have been published demonstrating the SWAT applications on sensitivity analyses, model calibration, hydrologic analyses, pollutant load assessment, and climate change impacts on hydrology and pollutant losses [35]. The key GIS input files to SWAT for this study included a 30 m digital elevation model (DEM) [36], 28.5 m land use/land cover [37], and Soil Survey Geographic (SSURGO) soil data at a scale of 1:24,000 [38]. The watershed was delineated into several subbasins based on DEM and the outlets selected within the watershed. Subsequently, the subbasins were partitioned into homogeneous units (hydrologic response units, HRUs) by setting threshold percentages of land use and soil type [30,39]. In this study, a threshold for a land use and soil type covering an area of 0% and 0%, respectively, within any given subbasin was applied in order to capture all the land use changes that occurred during the study period. This resulted in a total number of 1,465 HRUs in the watershed. Weather data (daily precipitation, minimum and maximum temperature) were obtained from Fayetteville Weather Station located approximately 25 km from the watershed. Other weather variables needed by the model (solar radiation, wind speed and relative humidity) were estimated using the weather generator built into the SWAT model.

The SWAT model has the ability to define specific types of manure and fertilizers by building fertilizer and manure components, such as fractions of mineral N (P), organic N (P), and a ratio of ammonium nitrate to mineral N in the SWAT fertilizer database. The pasture management information, including amount of litter and fertilizer application, timing of manure and fertilizer application, grazing intensity and dates were obtained from a detailed review of historical nutrient management plans and interviews with 63 out of 75 farmers in the watershed [40]. The baseline scenario consists of actual nutrient management and grazing management applied in the watershed during 1992–2004. The average litter application and approximate dates of application were 2,500 kg/ha applied on 30 April and 31 August. The manure excreted from grazing management ranged from 0.01–14.2 kg/(ha·day) for grazing days ranging from 11–365 days in the watershed during 1992–2004. Those management practices were implemented throughout the entire pasture area in the watershed. Detailed information of types of fertilizer and manure, the SWAT fertilizer database, and management practices and schedules for SWAT management files can be found in Chiang *et al.* [32].

Model calibration and validation were performed for monthly stream flow, total sediment (TS), total nitrogen (TN) and total phosphorus (TP) using the measured flow and water quality data collected at the Upper Moores Creek for the period January 1996–February 1999, January 2000–December 2003 and January 2006–December 2007. A total of 10 SWAT parameters were calibrated using Nash-Sutcliffe efficiency (NSE) [41] and coefficient of determination (R^2^) as the model performance criteria. Detailed information of calibrated SWAT parameters and performance of the SWAT model can be found in Chiang *et al.* [32].

### 2.3. BMP Scenarios

The watershed BMPs considered in this study were grouped into three categories: grazing and pasture management, vegetated filter strips, and nutrient management. These scenarios were based on detailed interactions with the watershed stakeholders and history of past BMPs implemented in the watershed [40]. Three grazing intensities were considered: (1) no grazing; (2) optimum grazing; and (3) overgrazing. The overgrazing application started on 30 September and lasted for 213 days until 30 April of the next year. The optimum grazing assumed that within 30 days the cattle should graze through the whole watershed and would stay for approximately 4–6 days in each pasture HRU [42]. This approach was similar to grazing operations reported in other watersheds located near the study area [43]. 

Vegetated filter strips (VFS) have been proven to be an effective management practice for trapping sediment and nutrients in field runoff [44,45,46], and reducing the transport of sediment and nutrients to down-gradient area [47,48]. Based on the worst condition of sediment delivery simulated in the previous study [49] and a method to design and estimate sediment removal from VFS by the Natural Resources Conservation Service [50], buffer strips with VFS ratios of 42 and 76 were simulated in this study. 

Nutrient management scenarios evaluated in this study included poultry litter application rates, litter characteristics, and application timing. The litter application rates evaluated were 1, 1.5 and 2 tons/acre in spring (applied on 30 April) and summer (31 August) to support growth of warm season grasses, and 2, 2.5 and 3 tons/acre in fall (15 October) to support growth of cool season grasses. For all application rates and timings evaluated in this study, two types of poultry litter were selected—normal poultry litter and alum-amended litter.

A total of 171 BMP combinations were simulated using the SWAT2009 model with dynamic land use changes during 1990–2007 in a previous study [32] and the performances of those BMP combinations in terms of pollutant reduction rates were further calculated for this study. It should be noted that the latest SWAT2009 model, which was publicly released in January 2010, has incorporated dynamic land use changes and modified simulation of vegetated filter strips. The model application simulating the impacts of dynamic land use changes concurrent with installation of conservation practices in the Lincoln Lake watershed can be found in Chiang *et al.* [32]. In order to evaluate the performance of the optimization models with different sets of BMP options, those 171 BMPs were grouped by litter application in spring (SP, a total of 54 BMPs) and summer (SU, a total of 54 BMPs), no grazing (NG, a total of 57 BMPs), optimum grazing (OG, a total of 57 BMPs), no buffer strips (VFS0, a total of 57 BMPs) and buffer strips with a ratio of 42 (VFS42, a total of 57 BMPs).

### 2.4. Multiobjective Genetic Algorithm Model

**Figure 2:**
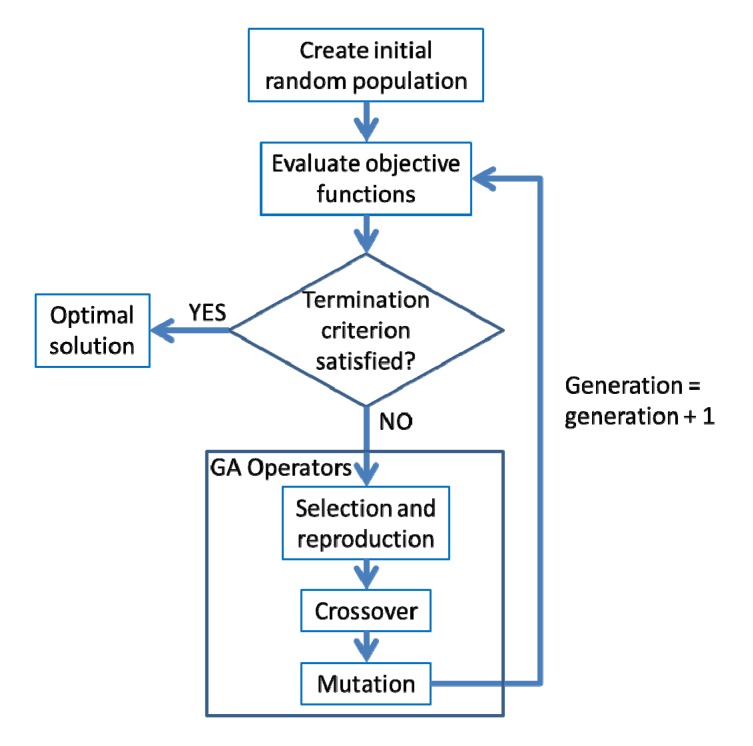
Overview of the GA method.

A genetic algorithm (GA) is a search technique to find solutions for optimization problems. Genetic algorithms are based on techniques inspired by evolutionary biology such as inheritance, selection, crossover and mutation. An algorithm is started with a set of solutions (chromosomes), called a population. The initial population of chromosomes is randomly generated for the given population size (Figure 2). Inheritance is the ability of the modeled object to mate, mutate and propagate the population as evolved solutions to a problem. A GA follows an iterated procedure (Figure 2). First, evaluate objective functions by computing a fitness value for each number of the population. Second, select a pair of chromosomes (parents) for mating (reproduction). During the selection process, the existing solutions from one population are taken into the mating pool and used to form a new population (children) based on their fitness; the higher the fitness of solutions are the more chance they have to reproduce. Third, the solutions in the mating pool then undergo the genetic operations: crossover and mutation. Crossover is a process that the new generation (child solutions) shares many of the positive characteristics of the parents, while mutation is a process that a bit in the solutions of a population is selected randomly and altered from its original state. The generational process is repeated until a termination condition (e.g., a solution is found that satisfies minimum criteria) has been reached. 

Multiobjective optimization problems have been evaluated in the hydrology/water quality field, where optimal decisions need to be taken between two or more conflicting objectives. Single-objective optimization yields a single optimal solution, while a multiobjective optimization produces a family of near-optimal solutions known as Pareto-optimal set. Deb *et al.* [51] concluded that the nondominated sorted genetic algorithm (NSGA-II) can search a larger number of variables and better spread of solutions than the strength Pareto evolutionary algorithm (SPEA-2) [52]. In this study, a total of 461 pasture HRUs are the variables for which the BMPs are to be searched to meet the two objective functions: (1) minimization of pollutant loading and (2) minimization of the pasture area that has BMPs implemented. The greater the pasture areas that have BMP, the less the polluatant loading. The two objective functions are mathematically expressed as follows, where f(*x*) denotes total pollution load and g(*x*) denotes the percentage of pasture area with BMPs implemented in total pasture lands. It should be noted that total nitrogen (TN) and total phosphorus (TP) losses were the two pollutants of concern for BMP implementation in this watershed:

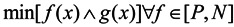
(1)

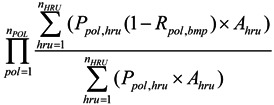
(2)

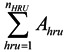
(3)
where *pol* is the number of pollutants of concern (*pol* = 1 for TN and *pol* = 2 for TP), *P*_pol, hru_ is the unit pollutant load from a *HRU*, *R*_pol, bmp_ is the pollutant reduction efficiency of BMP, *A* is the area of *HRU*.

A BMP tool was used to provide pollutant effectiveness for each BMP that can be implemented at a *HRU* scale in the watershed [22]. In a previous study, the SWAT model was run for those 171 BMP scenarios for dynamic land use and management practices during 1992–2007 with the corresponding historical weather [32]. The pollutant reduction efficiency was estimated by calculating the percentage reduction in the pollutant load for a BMP scenario compared with the baseline pollutant load. In order to narrow the search space for a given land use, seven allele sets of different BMP groups for pasture lands were created. Those allele sets for pasture lands are: a set of 171 simulated BMPs applicable to pasture HRUs (All), and other six sets of BMPs that contain no grazing (NG), optimum grazing (OG), spring litter application (SP), summer litter application (SU), no buffer strips (VFS0) and buffer strips with a VFS ratio of 42 (VFS42). During the optimization process, the algorithm first searches one BMP in the allele set for a pasture HRU. The estimation of the pollutant loading for the placement of that BMP in the selected pasture HRU is obtained from the BMP tool. An aggregated pollutant index (API), which is a product of the area-weighted pollutant reduction rates for TN and TP, and the total pasture lands that have BMP implemented were calculated for an estimation at the watershed scale.

Four parameters for a GA optimization are population size, number of generations, crossover rate and mutation probability. Population size determines the number of solutions considered for the evolutionary process. Crossover rate and mutation probability are critical in the optimization process in terms of creating a new set of child population which might be stronger than the parent population and eliminating the weaker individuals. The optimization process continues until a given number of iterations known as generations. Generally, the larger the population size, the more spread the solution space. Increasing the number of generations can also improve the performance of GA. However, it also increases the computing time to reach the near-optimal solution. 

### 2.5. Sensitivity Analysis and Estimation of GA Parameters

A sensitivity analysis of four GA parameters for different allele sets (BMP options) was performed to determine the influence of the parameters on the Pareto-optimal front and to identify the optimal parameter values. In order to evaluate the individual influence of a GA parameter on the Pareto-optimal front, one parameter (population size, number of generation, crossover and mutation probability) was changed at a time and the other parameters remained as default values (Table 1). The goodness of the Pareto-optimal front is determined subjectively as the closer the front gets to the origin, the better the solution is to minimize the two objective functions. When the front with a specific parameter value is closer to the origin, that value is then used for the optimization process. The aggregated pollutant index (API) and the percentage of pasture lands with BMPs implemented were estimated from equations (2) and (3). These two values are plotted against each other during the sensitivity analysis to obtain the optimal parameter values that have the Pareto-optimal front closest to the origin.

**Table 1:** Default and optimal GA parameters for different allele sets selected from sensitivity analysis.

Parameter	Population	Number of Generations	Crossover Probability	Mutation Probability
Default	100	1,000	0.9	0.0001
Optimal for different allele sets				
171 BMPs (All)	5,000	40,000	0.9	0.001
NG BMPs	3,000	40,000	0.5	0.001
OG BMPs	5,000	40,000	0.9	0.001
SP BMPs	3,000	40,000	0.7	0.001
SU BMPs	3,000	40,000	0.9	0.001
VFS0 BMPs	5,000	40,000	0.7	0.001
VFS42 BMPs	3,000	40,000	0.5	0.001

### 2.6. Targeting Method

The targeting method was chosen as a comparison of the selection and placement of BMPs from the GA optimization tool. The pasture HRUs were first ranked by the TN or TP losses. A single BMP scenario that has the greatest TN or TP reduction rate is implemented on the top ranked HRUs which accounted for 20%, 40%, 60%, 80% and 100% of the total pasture area. The pollutant losses from the entire pasture lands were calculated by summing up the pollutant losses from the HRUs that have the selected BMP scenario implemented and the current pollutant losses from the rest of pasture HRUs, and then divided by the total pasture area. One of the differences between GA optimization method and targeting method is that GA requires much longer computing time for a larger population size (>5,000) to get a wide range in solution space (*i.e.*, large number of choices for various pollutant reductions and areas under BMP combinations). Contrarily, the targeting method can provide a quicker solution by placing a BMP option on any pasture area that has relatively high TN or TP losses. In order to compare the performance of the optimization tool and the targeting method, the optimal percentage of BMP-implemented area that is identified by GA optimization was used to target the top ranked pasture HRUs which account for the same percentage of the area and calculate the pollutant losses from the pasture lands.

## 3. Results and Discussion

### 3.1. Sensitivity and Estimation of GA Parameters

**Figure 3:**
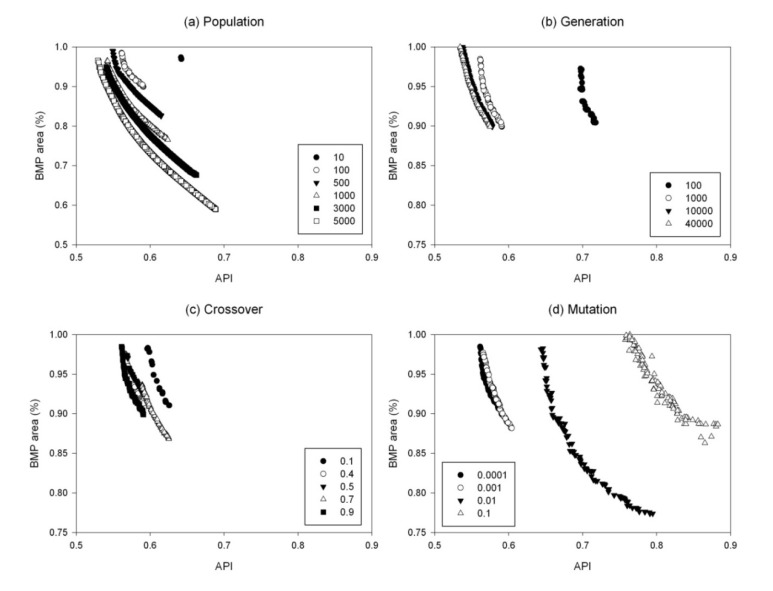
Pareto-optimal fronts for the sensitivity analysis of genetic algorithm (GA) parameters for the optimization model with a set of 171 BMP options (All).

**Figure 4:**
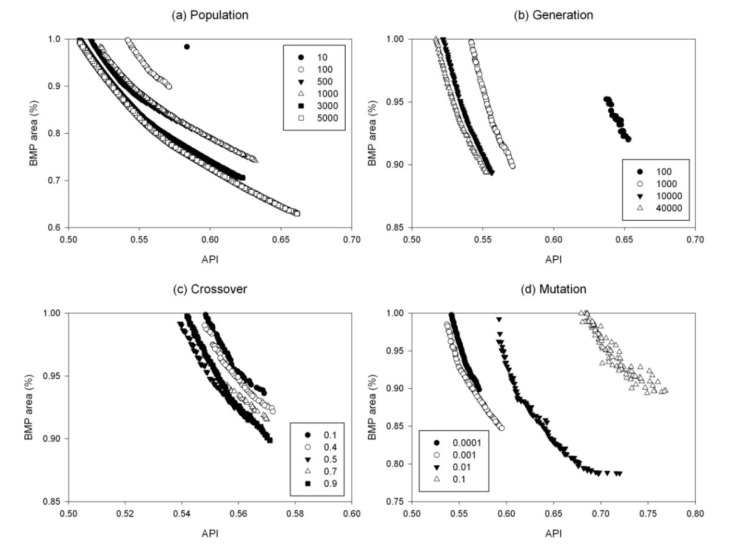
Pareto-optimal fronts for the sensitivity analysis of genetic algorithm (GA) parameters for the optimization model with a set of VFS42 BMP options.

The optimal GA parameters were selected using the sensitivity analysis of the optimal front. A total of seven sets of sensitivity analyses were performed for 171 BMPs. The sensitivity of GA parameters for the optimization model with a set of 171 BMP options (All), namely, population size, number of generations, crossover probability, and mutation probability, are shown in Table 1. Two GA parameters, population size and number of generations, can influence the computing time of the optimization. For example, when the population size increased from 100 to 5,000, the computation time increased from 10 min to 12 h for 1,000 generations. Similarly, the computation time increased from 10 min to 1 h when number of generation increased from 100 to 5,000. The maximum population size tested in this study was 5,000, with which the GA optimization tool could result in the most spread in the solution space in terms of the percentage of BMP-implemented pasture area for all different sets of BMP options. The sensitivity analysis results of the VFS42 BMP options (VFS42) and 171 BMP options (All) were selected for comparison (Figure 3 and Figure 4). It is because buffer strips are the most effective BMP in reducing pollutant losses and considerably greater pollutant reduction is expected if buffer strips with a VFS ratio of 42 are considered in the suite of BMP options.

As the population size increased from 10 to 5,000, more individuals were present during each evolution and there was a higher probability of obtaining a better offspring. Therefore, an improvement of the Pareto-optimal front that is getting closer to the origin was observed during the increases of population size. For the 171 BMP options, when population size further increased to 5,000 the individuals in the solution space had more freedom in terms of more spread of solutions compared to other population sizes (Figure 3a). Therefore, a population size of 5,000 was selected as an optimal value for the models with the sets of 171 BMP, OG BMP and VFS0 BMP options (Table 1). However, when only the BMPs with buffer strips of a VFS ratio of 42 were considered, an increase in population size from 3,000 to 5,000 did not show a considerable improvement in the Pareto-optimal front (Figure 4a). It should be noted that during sensitivity analysis, only one GA parameter of concern was changed and the other three parameters were fixed at their default values. Therefore, a population size of 5,000 might require more generations (>1,000) for the individuals to show a considerable change in the objective functions. However, as the number of generations increases, the computing time would also increase. Therefore, a population size of 3,000 was selected as an optimal value for the models with the sets of NG BMP, SP BMP, SU BMP and VFS42 BMP options.

Similar to the population size, an increase in the number of generations can lead the Pareto-optimal front closer to the origin and a better optimal solution can be found. The larger the number of generations is, the better the fittest individuals for reproduction can be selected. The Pareto-optimal front greatly improved when the number of generations increased from 100 to 1,000, while there was no considerable change between 10,000 and 40,000 generations (Figure 3b and Figure 4b). A similar result was observed for the models with other sets of BMP options. It was noticed that the Pareto-optimal front considerably improved as the number of generations increased. However, a model run with a combination of the population size of 3,000 or 5,000 and the 40,000 generations will take much more computation time. Therefore, 10,000 generations were used in the final optimization model for all sets of BMP options.

Unlike the population size and the number of generations, an increase in crossover probability did not always result in a better Pareto-optimal front. For example, the solutions of the model with the 171 BMP options improved when the crossover probability increased from 0.1 to 0.4, but the front moved away from the origin when it further increased to 0.5 and 0.7 (Figure 3c). The optimal solution was found when the crossover probability was 0.9, indicating that the higher crossover probability leads to faster convergence. The same optimal crossover probability (0.9) was found for the models with the sets of OG BMP and SU BMP options, while other models had different optimal values (0.5 and 0.7) (Table 1). 

No consistent pattern in the shift of the Pareto-optimal front was found for the mutation probability. For both the models with sets of 171 BMP and VFS42 BMP options, a slightly higher mutation probability (0.001) than the default value (0.0001) made the Pareto-optimal front move toward the origin (Figure 3d and Figure 4d). Further increases in the mutation probability (0.01 and 0.1) resulted in a dramatic deterioration in the performance. Similar results were found in other models with different sets of BMP options, and the optimal value for mutation probability (0.001) was used in the final BMP optimization model for the models with all different sets of BMP options.

**Figure 5:**
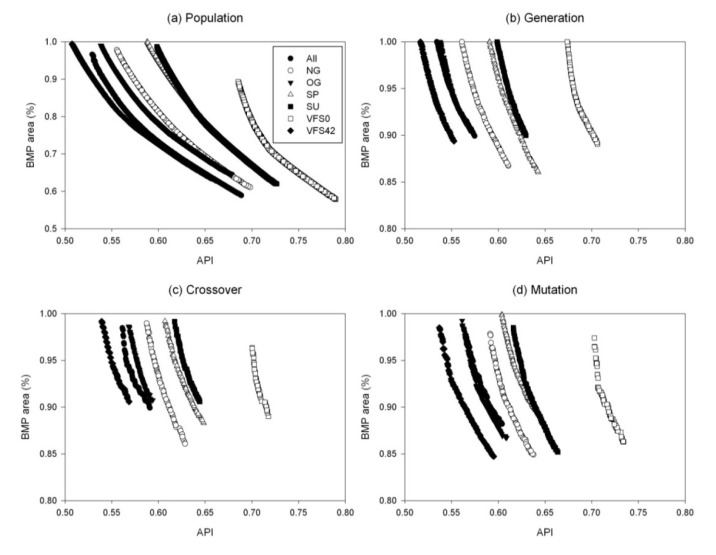
Comparison of the Pareto-optimal fronts for models with different BMP options using their optimal GA parameters.

An interesting result was observed when comparing the Pareto-optimal fronts for these models with different BMP options using their optimal GA parameters (Table 1 and Figure 5). Generally, the Pareto-optimal front of the model with VFS0 BMP options was the farthest from the origin, indicating that the solutions obtained from the BMPs with no buffer strips were greatly limited in finding an optimal solution in terms of minimizing the BMP-implemented area and minimizing the nutrient losses. The best performance of the Pareto-optimal front was observed for the model with the VFS42 BMP option, followed by two models with NG and OG BMP options, and the other two models with SP and SU BMP options. This can be explained as buffer strip is the most effective management practice in reducing pollutant losses and most of the nutrient losses come from land application than grazing management.

### 3.2. Performance of the Optimization Tool and the Targeting Method

**Figure 6:**
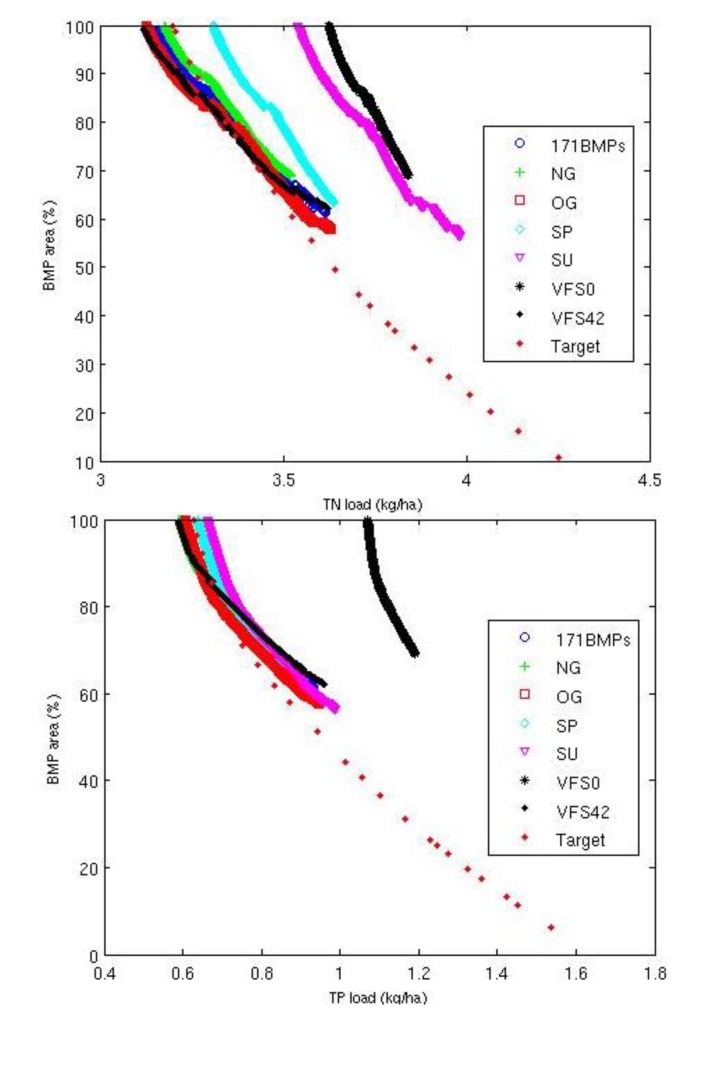
Comparison of the Pareto-optimal fronts of different optimization models after the final generation and the solutions obtained from the targeting method for total nitrogen (TN) and total phosphorus (TP) reduction.

After assessing the sensitivity analysis for the GA parameters, various final values of the GA parameters were applied to each model to search for the optimal BMP solutions (Table 1). The optimized results were then compared to the solutions obtained using the targeting method (Figure 6). The average annual area-weighted baseline loadings from the pasture lands were 4.55 kg/ha and 1.66 kg/ha for total nitrogen (TN) and total phosphorus (TP), respectively. The optimization provided 3,000–5,000 solutions during each generation depending on the BMP options were considered. All the solutions in the last generation (10,000th) were presented in the Pareto-optimal front (Figure 6), and the optimal solution was found as median of the range of the pollutant loads and BMP-implemented area for each BMP option (Table 2). For the optimization model with the 171 BMP option, the optimal solution resulted in nutrient losses of 3.39 kg/ha (25.54% reduction) and 0.74 kg/ha (55.12% reduction) for TN and TP, respectively when various BMPs are implemented in 77% of the entire pasture areas. The optimal solution for the model with the NG BMP options showed that greater nutrient reductions would be found if BMPs are installed in more than 77% of the pasture areas. Generally, there is no significant difference among the solutions for the models with 171 BMP options and different grazing options (Figure 6). However, even more pasture areas having BMPs with only spring or summer litter application implemented, less nutrient reductions could be found in the watershed compared with the 171 BMP option. When only summer litter application was considered, much less TN reduction would be achieved compared to the solutions with SP BMP options. Similarly, the optimal solution for the model with the VFS0 BMP option was 3.75 kg/ha and 1.11 kg/ha of TN and TP loads, respectively, with various BMPs implemented in 82% of the pasture areas. It showed that even if more pasture lands have BMPs implemented, less pollutant reduction would be achieved if buffer strips were not among the BMP options considered. Those observations were consistent with the observation in the sensitivity analyses for different models (Figure 5) that the Pareto-optimal fronts of the models with sets of VFS0 and SU BMP options were away from the origin, indicating that the low pollutant reduction rates of the selected BMPs themselves can affect the overall reduction obtained from the optimization. 

A total of 461 pasture HRUs were ranked by the pollutant losses. The BMPs that resulted in the greatest TN reduction rate are the BMP combination of buffer strips (*VFS ratio* = 42 or 76) and no litter application. However, use of fertilizer or manure to support plant growth is needed and generally found in the watershed. Except the no litter application, the litter types, application timing and amount can be optimized. Therefore, an optimal suite of BMPs (scenario 81) to reduce TN losses, which has the TN reduction rate of 28.33%, is a combination of buffer strips (*VFS ratio* = 42), optimum grazing and 1 ton/acre litter application in spring. While the optimal suite of BMPs (scenario 59) to reduce TP losses with a TP reduction rate of 62.15% is the combination of buffer strips (*VFS ratio* = 42), no grazing and 1 ton/acre alum-treated litter application in spring. By using the targeting method, the least annual TN and TP losses from pasture area that would be seen were 3.26 kg/ha and 0.63 kg/ha if the optimal suites of BMPs were adopted in all pasture lands (Figure 6). When 50% of the pasture lands have the optimal BMPs implemented, the TN and TP loads could reduce to 3.65 kg/ha (19.15% reduction) and 0.96 kg/ha (41.98% reduction), respectively. While a greater reduction in the pollutant losses can be obtained from BMP optimization, the computation time requirement for the optimization is considerably longer, when using a 3,000–5,000 population size and 10,000 generations, than the targeting method, which is simply a ranking of pollutant losses from HRUs. However, the targeting method does not compare interactions among BMPs, and adoption of a single suit of BMPs throughout the pasture lands may not be practical due to various land characteristics or farmers’ choices of BMPs.

**Table 2:** Solutions which are medians of the range of pollutant loads and BMP-implemented area for different optimization models.

BMP Options	TN Load (kg/ha)	BMP Area(%)	TP Load (kg/ha)	BMP Area(%)
ALL	3.39	0.77	0.74	0.77
NG	3.36	0.82	0.70	0.82
OG	3.41	0.75	0.74	0.75
SP	3.51	0.78	0.75	0.78
SU	3.77	0.74	0.79	0.74
VFS0	3.75	0.82	1.11	0.82
VFS42	3.38	0.77	0.77	0.77

Note: ALL denotes all 171 BMP options; NG denotes the BMP options containing only BMPs with no grazing management; OG denotes the BMP options containing only BMPs with optimum grazing management; SP denotes the BMP options containing only BMPs with spring litter application; SU denotes the BMP options containing only BMPs with summer litter application; VFS0 denotes the BMP options containing only BMPs with no buffer strips; VFS42 denotes the BMP options containing only BMPs with buffer strips with a VFS ratio of 42.

### 3.3. Comparison of Selection and Placement of BMPs

**Figure 7:**
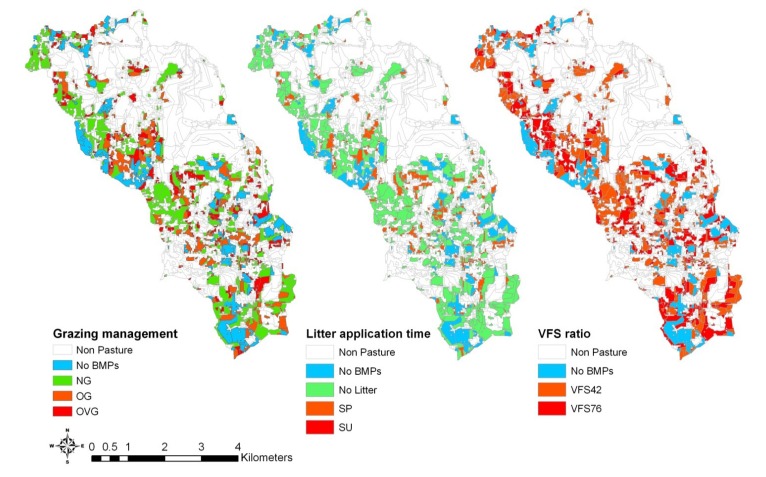
Distribution of BMPs selected by the optimization model with the 171 BMP options on 77% of the pasture lands in the watershed.

The optimal solution for the model with 171 BMP options was distributed throughout the watershed (Figure 7). The distribution of those optimal BMPs were presented into three maps with different legend categories, which are grazing management, litter application timing and buffer strips. The blue areas denote no BMPs were selected for those areas, which account for 23% of the total pasture area. Among those selected BMPs, no BMPs with fall litter application and no buffer strips were selected by the optimization tool. It was observed that no grazing management, no litter application and buffer strips with a VFS ratio of 42 were frequently selected by the optimization tool, indicating this BMP combination can reduce nutrient losses more effectively than other BMP combinations. From the perspective of a watershed manager, those extreme management practices can reduce the greatest pollutant losses without considering the crop yields. If this optimal solution is adopted in the watershed, TN and TP losses can be expected to reduce by 25.5% and 55.1%, respectively. However, various BMP combinations can be designed as BMP options for optimization tool to assess the pollutant reduction by optimal practical or farmers-preferable management practices. 

**Figure 8:**
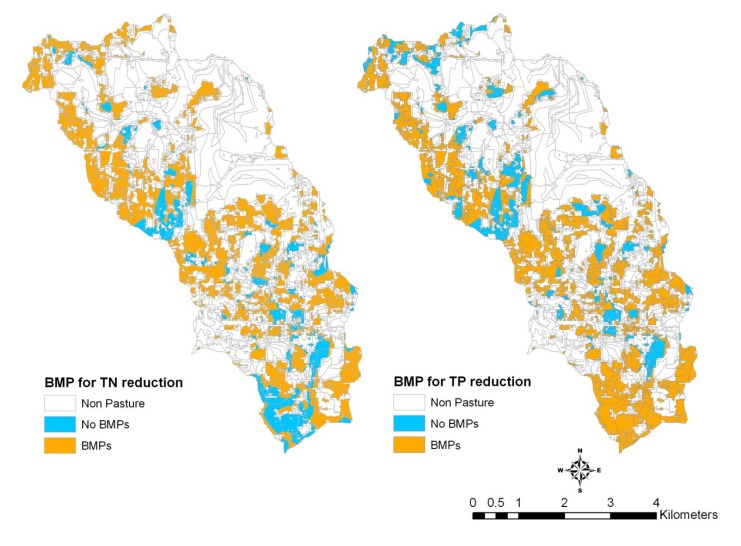
Distribution of BMPs selected by the targeting method on 77% of the pasture lands in the watershed.

Compared to the distribution of the BMP-implemented area from the optimization tool, a slightly different distribution of the selected BMP in the watershed by using the targeting tool was observed (Figure 8). It should be noted that the total percentage of original pasture lands that have BMP implemented is the same as the solution from the optimization tool. The BMP combination (BMP 81) that has the greatest TN reduction rate was selected as the optimal BMP combination to reduce TN losses. Likewise, the BMP combination (BMP 59) was selected as the optimal BMP combination to reduce TP losses. The blue areas denote no BMPs were selected for those areas. It was observed that the distributions of targeted area for reducing TN and TP losses were different, indicating that a proper BMP combination needs to be adopted to reduce both pollutants of concern. 

**Figure 9:**
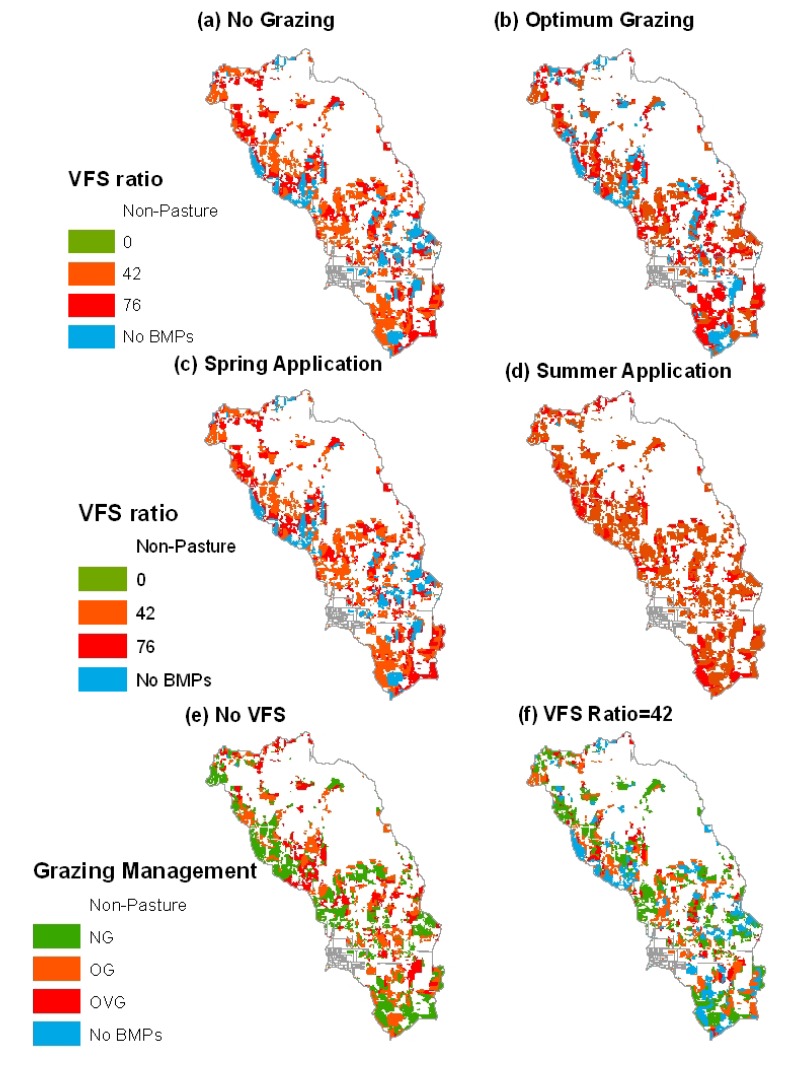
Distribution of selected BMPs from different optimization models to meet the same pollutant reduction of the model with the 171 BMP options.

In order to compare the performance of optimization models with different BMP options, various solutions that meet either the optimal pollutant reduction or BMP-implemented area by the model with 171 BMP options were selected (Table 3 and Figure 9). Both solutions from the models with NG and OG BMP options were able to reduce TN losses to 3.39 kg/ha, which was the same as the optimal TN reduction of the model with 171 BMP options (Figure 9a,b). Those BMP combinations were grouped by no BMP, no buffer strips, buffer strips with a ratio of 42 and with a ratio of 76. The selected NG BMPs were implemented on 80% of the pasture lands, while the selected OG BMPs were implemented on 77% of the pasture lands, which is the same as the optimal BMP-implemented area for the model with 171 BMP options. 

When only BMP combinations that consist of litter application in spring were selected for the optimization model, more pasture lands (89%) are needed to achieve the same pollutant reduction from the optimization model with the 171 BMP options (Figure 9c). It was observed that even all the pasture lands have BMPs that consist of litter application in summer, the pollutant losses with 3.54 kg/ha of TN load and 0.66 kg/ha of TP load were relatively higher than the pollutant reduction from the optimal solution of the model with 171 BMP options (Figure 9d). It indicated that BMPs with the litter application in summer should be avoided. Similarly, when only the BMPs with no buffer strips were evaluated, less effectiveness of those BMPs could be expected even if they were adopted on all pasture lands (Figure 9e). The higher pollutant loads resulted by implementing VFS0 BMPs throughout the pasture lands than by implementing VFS42 BMPs indicated that buffer strips are most effective management practices to reduce pollutant losses. The effectiveness of buffer strips with a ratio of 42 indicated that fewer pasture areas (75%) are needed to have BMPs implemented to improve water quality (Figure 9f).

**Table 3:** Solutions from other models which at least meet the same pollutant reduction of the model with 171 BMP options.

BMP options	TN Load (kg/ha)	BMP Area(%)	TP Load (kg/ha)	BMP Area(%)
Baseline	4.55	0.00	1.66	0.00
ALL	3.39	0.77	0.74	0.77
NG	3.39	0.80	0.72	0.80
OG	3.39	0.77	0.73	0.77
SP	3.39	0.89	0.68	0.89
SU	3.54	1.00	0.66	1.00
VFS0	3.62	1.00	1.07	1.00
VFS42	3.39	0.75	0.78	0.75

## 4. Conclusions

Many studies have been conducted to optimize the selection and placement of BMPs to economically reduce pollutant loads from watersheds by using plan- or performance-based methods. The objectives of this study were to: (1) compare the selection and placement of BMPs using a genetic algorithm (GA) optimization and a targeting method; (2) evaluate the impacts of various BMP options

on the optimal solutions from optimization. Two objective functions were used to minimize the TN and TP losses and the BMP-implemented pasture area. It was found that optimization required much longer computation time than the targeting method to obtain a more spread of solutions. The solutions obtained from the optimization tool were optimal for both reducing TN and TP losses by placing BMPs in the same pasture areas, while the targeting method focused on reducing one individual pollutant loading at a time by placing a single suite of BMPs in different areas, which may not be practical due to various land characteristics or farmers’ choices of BMPs. Overall, when using the targeting method more pasture areas are needed to have BMPs implemented in order to achieve the same pollutant reductions that result from the optimal BMPs selected by optimization.

A total of 171 BMP scenarios were grouped by no grazing (NG), optimum grazing (OG), spring litter application (SP), summer litter application (SU), no buffer strips (VFS0) and buffer strips with a VFS ratio of 42 (VFS42) as various sets of BMP options for evaluating their impacts on the optimal solutions from the optimization model. The results showed that limiting the BMP options to certain BMPs, such as buffer strips with a VFS ratio of 42, could result in greater pollutant reductions within smaller pasture areas managed with BMPs. However, when only summer litter application or no buffer strips are considered during optimization and the optimal BMPs are implemented in the entire pasture areas, they still resulted in greater pollutant losses than the solutions from the model with 171 BMP options. Therefore, it is essential to carefully select the BMP options for optimization in order to obtain more effective solutions in minimizing pollutant losses and BMP-implemented area in a watershed. Moreover, for a more comprehensive evaluation of selection and placement of BMPs in a watershed, other pollutants of concerns, and cost and maintenance of selected BMPs options should be taken into consideration when applying this evaluation framework. 
